# Among the Graduates of the Ontario Veterinary College

**Published:** 1898-07

**Authors:** 


### Among the Graduates of the Ontario Veterinary College.

Dr. James A. Waugh, of the class of 1882, early sought the experiences of a military life as a veterinarian, but, tiring of the lack of official recognition, sought in the "Smoky City" the pleasures and profits of a civil practice.

Dr. John A. Bell, of the class of 1880, was an earnest seeker after sanitary science and police, and as naturally drifted into official work in the "Empire State."

Dr. Judson Black, of the class of 1894, was a strong advocate of the necessity of a proper knowledge of horseshoeing to make a well-equipped veterinarian.

Dr. F. A. Balser, of the class of 1885, has filled the rôle of State veterinarian for the "Hoosier State."

Dr. John P. Bond, of the class of 1873, early sought association with the work of the Bureau of Animal Industry.

Dr. W. G. Benner, of the class of 1890, finds much pleasure and enjoyment in the raising of German hares.

Dr. Tait S. Butler, of the class of 1885, is an ardent swine-raiser, and prophesies the early transition of the Southern razor-back to a full-fledged "Chief, I know" of the West.

Dr. R. W. Carter, of the class of 1883, finds no animal to so well fill his eye for beauty as the thoroughbred.

Dr. John T. Claris, of the class of 1882, set his spurs for the erection of one of the largest and most commodious hospitals in the "Queen City of the Lakes."

Dr. T. Bent Cotton, of the class of 1882, was a prime mover and officer in the National Veterinary Association of the United States.

Dr. Anderson Crowforth, of the class of 1891, is a noted admirer of the standard-bred trotter, and takes a keen interest in the wonderful performers of the present day.

Dr. R. R. Dinwiddie, of the class of 1886, finds the work of the State experiment stations congenial to his tastes.

Dr. F. H. P. Edwards, of the class of 1889, is an earnest association advocate.

Dr. Charles Elliott, of the class of 1870, continues to take a strong interest in the advancement of his alma mater.

Dr. Samuel Foelker, of the class of 1879, is one of the most earnest and zealous graduates of the college. He never tires in well-doing for the welfare of his patients.

Dr. E. A. A. Grange, of the class of 1873, was one of Ontario's most earnest students and was early called to impart his knowledge at the Guelph College, and now fills the rôle of Dean of the Veterinary Department of the Detroit College.

Drs. H. and J. Heckenberger, of the classes of 1878 and 1879, respectively, are among the Keystone State's most successful practitioners.

Dr. J. Z. Hillegass, of the class of 1890, is a great admirer and driver of the American trotter.

Dr. T. D. Hinebauch, of the class of 1887, has contributed much to the study of millet as an equine food.

Dr. W. J. Hinman, of the class of 1875, is one of the foremost promoters of the Manitoba Veterinary Association.

Dr. Albert Drinkwater, of the class of 1874, takes an active interest in his county veterinary association of New York State.

Dr. J. T. Duncan, of the class of 1872, has never wandered far away from the fold, and it would not seem like the old place to many to find him absent from old "Toronto's" walls on their annual pilgrimages to alma mater.

Dr. J. H. Frinck, of the class of 1879, for a number of years took much interest in the U. S. V. M. A. Association affairs for the Province of New Brunswick.

Dr. F. C. Grenside, another of the class of 1879, is an expert horseman, well versed in the judging and breeding of horses.

Dr. C. E. Hollingsworth, of the class of 1887, has proved a strong association member in the "Prairie State" organization.

Dr. Joseph Hawkins, of the class of 1871, has for many years been one of the most devoted members of the profession in the "Wolverine State."

The profession in Virginia had no more sincere and earnest worker among its members than in the late Dr. W. H. Harbaugh, a member of the class of 1885. He laid down his life for the advance of his chosen profession.

Dr. J. B. Irons, of the class of 1883, is an ardent worker in his calling and takes much interest in association affairs in the Keystone State.

Dr. Wilson Huff, of the class of 1885, never rests in his determination that veterinary sanitary police measures will prevail throughout the "Empire State."

Dr. William R. Howe, of the class of 1883, has long yearned for the U. S. V. M. A. to visit Dayton, Ohio, on one of its annual conventions.

Dr. J. Rein Keelor, another of the class of 1883, has long yearned for journalistic honors in the equine press.

Dr. Thomas King, of the class of 1889, was one of the promoters of the now defunct Ohio Veterinary College at Cincinnati, Ohio.

Dr. D. McIntosh, of the class of 1869, has contributed several publications to veterinary literature; they have been prepared and written in a popular way.

McEver Brothers, of the classes of 1877, 1890, and 1892, respectively, have won fame and fortune in the "Windy City."

Dr. C. C. McLean, of the class of 1883, is very active in promoting safeguards around the production and sale of milk, and at Meadville, Pa., has established one of the best systems extant.

Dr. W. A. Meredith, of the class of 1884, has placed himself in line for future membership in the Keystone State organization, and is destined to be a worker in the ranks.

Dr. J. V. Newton, of the class of 1878, has wandered into the field of proprietary remedies, a field of danger and disappointment.

Dr. Claude D. Morris, of the class of 1888, has proved a faithful and diligent member of the flock, and is to-day an ardent worker for every veterinary reform.

Dr. L. A. Merrillat, of the class of 1888, is the able assistant of Prof. M. H. McKillip, of Chicago, and a member of the Faculty of the McKillip Veterinary College.

Dr. W. T. Monsarrat, of the class of 1889, is another member of the American colony in Hawaii. He is active in seeking legislative protection for graduates in that island.

Dr. J. H. Oyler, of the class of 1887, has an eye ever vigilant after those who would violate State laws, and has been an earnest supporter of State veterinary legislation in Pennsylvania.

Dr. Charles A. Sankey, of the class of 1894, is a frequent contributor to agricultural journals.

Dr. S. Sisson, of the class of 1891, remains a faithful worker in the ranks of the instructors of his alma mater.

The classes of 1871, 1877, 1884, and 1892 have each a representative as a graduate of the Somerville family of Buffalo, N. Y.

Dr. M. Stalker, at the head of the Veterinary Department of the Iowa Agricultural College at Ames, represents the class of 1877.

Dr. Thomas M. Sweeny, for several years Secretary of the Virginia Veterinary Association, is a graduate of the class of 1895.

No more faithful teacher and representative of the best interests of a college ever lived than Dr. C. H. Sweetapple, of the class of 1869, who has given long years of service to the Ontario Veterinary College.

The Drs. Sutterby, of Batavia, N. Y., represent respectively the classes of 1878, 1887, and 1889. They were among the early supporters to the movement that led to the formation of the present New York State Association.

Dr. S. E. Weber, of the class of 1884, still continues to suffer from the results of the injuries to his limbs, and is only able to move around by the aid of crutches.

The Wende brothers represent the classes of 1882, 1884, and 1886. Dr. John Wende, of Buffalo, is one of the most successful practitioners of that city and a contributor to the State Association programme and the JOURNAL'S columns.

